# Loss of Prolyl Carboxypeptidase in Two-Kidney, One-Clip Goldblatt Hypertensive Mice

**DOI:** 10.1371/journal.pone.0117899

**Published:** 2015-02-23

**Authors:** Nadja Grobe, Orly Leiva, Mariana Morris, Khalid M. Elased

**Affiliations:** 1 Department of Pharmacology and Toxicology, Boonshoft School of Medicine, Wright State University, Dayton, Ohio, United States of America; 2 College of Osteopathic Medicine, Nova Southeastern University, Fort Lauderdale, Florida, United States of America; Cedars-Sinai Medical Center, UNITED STATES

## Abstract

It is well documented that angiotensin (Ang) II contributes to kidney disease progression. The protease prolyl carboxypeptidase (PRCP) is highly expressed in the kidney and may be renoprotective by degrading Ang II to Ang-(1-7). The aim of the study was to investigate whether renal PRCP protein expression and activity are altered in two-kidney, one-clip (2K1C) Goldblatt hypertensive mice. Left renal artery was constricted by using 0.12 mm silver clips. Blood pressure was measured using telemetry over the eleven weeks of study period and revealed an immediate increase in 2K1C animals during the first week of clip placement which was followed by a gradual decrease to baseline blood pressure. Similarly, urinary albumin excretion was significantly increased one week after 2K1C and returned to baseline levels during the following weeks. At 2 weeks and at the end of the study, renal pathologies were exacerbated in the 2K1C model as revealed by a significant increase in mesangial expansion and renal fibrosis. Renal PRCP expression and activity were significantly reduced in clipped kidneys. Immunofluorescence revealed the loss of renal tubular PRCP but not glomerular PRCP. In contrast, expression of prolyl endopeptidase, another enzyme capable of converting Ang II into Ang-(1-7), was not affected, while angiotensin converting enzyme was elevated in unclipped kidneys and renin was increased in clipped kidneys. Results suggest that PRCP is suppressed in 2K1C and that this downregulation may attenuate renoprotective effects via impaired Ang II degradation by PRCP.

## Introduction

There are 26 million adults with chronic kidney disease (CKD) in the United States and the number of those affected continues to increase [1]. The activation of the renin angiotensin system (RAS) and the elevated formation of the vasoconstrictor angiotensin (Ang) II both contribute to renal pathophysiology by stimulating pathways that lead to aldosterone release, water retention, vasoconstriction, fibrogenesis, and inflammation [2–4]. There is emerging evidence that Ang II and Ang-(1–7) have counter-regulatory roles. While Ang II functions as a potent vasoconstrictor and is implicated in the pathophysiology of various kidney diseases, Ang-(1–7) protects against renal damage and cardiovascular disease (5–8). Indeed, treatment or chronic infusion with Ang-(1–7) results in vasodilation, antiproliferation, antihypertrophy and antifibrosis mediated through binding of the heptapetide to the Mas receptor [5–12]. Angiotensin converting enzyme 2 (ACE2) converts Ang II to Ang-(1–7) [13]. ACE2 has been shown to be cardio- and renoprotective in various animal models of metabolic and cardiovascular diseases [14–26]. Notably, ACE2 deficient mice exhibit a normal phenotype at baseline as well as unaltered levels of Ang II and Ang-(1–7) in the kidney, heart, and plasma suggesting the presence of alternative pathways for peptide formation [5,14,17,27–32].

In addition to ACE2, Ang-(1–7) can be formed by prolyl endopeptidase (PREP) [32,33], prolyl carboxypeptidase (PRCP) [34], neprilysin (NEP) [33,35], thimet oligopeptidase [36] and neurolysin [36]. Recent work in our laboratory using novel mass spectrometric techniques for the characterization of RAS enzymes demonstrated that both ACE2 and PRCP contribute equally to renal Ang II degradation to Ang-(1–7) [37,38]. PRCP, also known as angiotensinase C (EC 3.4.16.2), was first isolated from human kidney, urine and leucocytes [39]. PRCP is an exopeptidase that catalyzes the cleavage of C-terminal peptide bonds with proline in the penultimate position. Different from ACE2, PRCP accepts also Ang III as a substrate producing Ang-(2–7) [40]. Although the C-terminal peptide sequence of Ang II and Ang III is identical, PRCP hydrolyzes Ang III faster than Ang II. While not much is known about the biological effects of Ang-(2–7) in humans, Ang III shares similar physiological activities with Ang II suggesting it may be equally or even more important than Ang II in some actions, e.g aldosterone or vasopressin release and blood pressure regulation [41–45].

The optimal PRCP enzyme activity naturally occurs at acidic pH ≤ 6. However, PRCP also exerts activity at neutral pH [39,46]. PRCP is localized in the kidney to the tubular apical membrane [47]. Originally, PRCP has been identified as soluble and lysosomal enzyme, and recent studies demonstrated membrane-bound PRCP [34,46–48]. Its known substrates are Ang II, Ang III, plasma prekallikrein, bradykinin, and α-melanocyte-stimulating hormone, suggesting a major role of PRCP in the regulation of vascular function, blood pressure, inflammation, food intake, and angiogenesis [46,49–53]. Indeed, PRCP deficient mice present with vascular dysfunction, oxidative stress, and reduced body weight [47,49]. A recent study found elevated plasma PRCP levels in diabetic and obese patients [54]. Additionally, PRCP E112D polymorphisms have been associated with decreased PRCP gene expression, hypertension, and preeclampsia [55,56]. However, the role of PRCP in renal physiology and pathophysiology has not been investigated before.

Therefore, the effects of chronic renal injury on the expression of PRCP was examined in clipped and contralateral, unclipped kidneys of mice with renovascular hypertension induced by clamping of the renal artery [57]. This approach is known as the two-kidney, one-clip (2K1C) Goldblatt (2K1C) model. The 2K1C model mimics renal artery stenosis in humans which has been shown to induce secondary hypertension and ischemic changes in the affected kidney [58]. The studies described herein aid toward dissecting the role of hypertension, hypoperfusion and elevated Ang II levels on renal PRCP in the 2K1C model of renovascular hypertension.

## Materials and Methods

### Animals

Male C57BL/6 mice were housed at 22°C under standard 12 hour light/12 hour dark conditions with *ad libitum* access to water and standard chow (Harlan Teklad, Madison, WI, USA). All experimental protocols were approved by WSU Animal Care and Use Committee (protocol number: AUP 937). All surgery was performed under 2% isoflurane anesthesia, and all efforts were made to minimize pain and suffering.

### Blood pressure, heart rate and locomotor activity measurements using radiotelemetric probes

Mice were anesthetized with 2% isoflurane. An incision was made along the neck. Using blunt dissection the left common carotid artery was located followed by clamping and cannulation. A sterile radiotelemetric catheter, model TA11PA-C10 (Data Sciences International, St Paul, MN, USA), was inserted into the carotid artery and tied in place. The body of the transmitter was inserted into a pouch below the skin on the right flank. The skin was closed using 5.0 sterile sutures (Arosurgical, Newport Beach, CA, USA). Carprofen (5 mg/kg; Sigma-Aldrich, St. Louis, MO, USA) was given SC immediately and 24 hour later for post-operative analgesia. Animals were allowed to recover for one week before blood pressure, heart rate and locomotor activity were recorded.

### Goldblatt Two-Kidney, One-Clip (2K1C) Surgery

Mice were anesthetized with 2% isoflurane and the kidney was visualized through a flank incision. A u-shaped silver clip (Exidel SA, Delemont, Switzerland) was inserted onto the left renal artery leaving a 0.12 mm gap for ischemic blood flow. Carprofen (5 mg/kg, SC) was given immediately and 24 hour later for post-operative analgesia.

### Kidney histology and immunohistochemistry

Kidneys were collected from mice anaesthetized by ketamine/xylazine (100:8 mg/kg, I.P.) and perfused transcardially with cold PBS followed by 4% paraformaldehyde. For histology, paraffin sections (4 μm) were stained with periodic acid-Schiff (PAS) and Masson’s trichrome (AML Laboratories, Baltimore, MD, USA) and examined under light microscopy. For immunofluorescence, paraffin sections were deparaffinized and incubated at 95°C for 30 min in 10 mM sodium citrate pH 6. Sections were then incubated with 10% horse serum diluted in 0.3% Triton PBS followed by primary antibody rabbit anti-PRCP (1:50 dilution, Biorbyt cat # orb13658, San Francisco, CA, USA) and goat anti-nephrin (1:200 dilution, R&D cat # AF3159, Minneapolis, MN, USA) diluted in 0.1% Triton PBS containing 5% horse serum. Sections were then incubated with donkey anti-rabbit Alexa-568 (Life Technologies, Grand Island, NY, USA) or donkey anti-goat Alexa-488 (Jackson ImmunoResearch Laboratories, West Grove, PA, USA) diluted 1:100. Sections were cover slipped using Vectashield hard set mounting medium (Vector Laboratories, Burlingame, CA, USA). Images were acquired with an Olympus FV300 confocal microscope (Olympus, Center Valley, PA, USA).

### Western Blot

Kidneys were quickly collected, flash frozen in liquid nitrogen and homogenized with 1.4 mm ceramic beads in complete lysis M-EDTA free buffer (Roche Diagnostics, Mannheim, Germany) supplemented with 2.5 mM PMSF using a Precellys24 bead homogenizer (Bertin Technologies, Montigny-le-Bretonneux, France). Kidney homogenates were centrifuged at 10,000 x g for 10 minutes at 4°C to remove cellular debris. Total protein content was determined using bovine serum albumin as standard and BioRad reagent (BioRad, Hercules, CA, USA). Kidney homogenates (60 μg protein) were loaded to a 10% SDS-PAGE gel and transferred to a 0.2 μm PVDF membrane (Millipore, Billerica, MA, USA). Membranes were probed with goat anti-PRCP (1:500 dilution, Biorbyt cat # orb13658, San Francisco, CA, USA), rabbit anti-PREP (1:2000 dilution, Abcam cat # ab58995, Cambridge, MA, USA) or goat anti-ACE (1:200, Santa Cruz Biotechnology, Inc., cat # sc-12184, Dallas, TX, USA) followed by incubation with horseradish peroxidase conjugated donkey anti-rabbit (1:3000 dilution, Abcam) or donkey anti-goat (1:5000 dilution, Santa Cruz Biotechnology). Bands were detected using SuperSignal chemiluminescent substrate (Pierce, Rockford, IL, USA) and visualized with a ChemiDoc MP imaging system (BioRad, Hercules, CA, USA) at ACE 195 kDa, PREP 81 kDa, PRCP 56 kDa. Protein intensity was normalized to ß-actin (Sigma, St. Louis, MO, USA) and analyzed using Imaging Lab 4.0 software (BioRad, Hercules, CA, USA).

### Renal PRCP and renin enzyme activity

Ang-(1–7) formation from Ang II was assessed using matrix assisted laser desorption ionization (MALDI) time-of-flight (TOF) based enzyme assays as previously described with some modifications [37,38]. For the *in situ* PRCP enzyme assays, consecutive tissue sections (12 μm) were prepared from fresh frozen kidneys and incubated with 1 mM Ang II at 37°C for 5 min. For the *in vitro* PRCP enzyme assays, kidney homogenate from whole kidney (20 μg protein) was incubated at 37°C in 50 mM sodium citrate buffer pH 5 containing 5 mM ZnCl_2_, 150 mM NaCl_2_, 10 μM lisinopril and 0.1 mM Ang II. The enzyme reaction was terminated by addition of 1% trifluoroacetic acid (TFA). Renin activity was measured as described before with some modifications [59]. Kidney homogenate from whole kidney (20 μg protein) was incubated at 37°C in 50 mM MES buffer pH 6.75 containing 1 mM EDTA, 100 μM peptidase inhibitors (thiorphan, 4-aminophosphonobutyric acid, Cpp-AAF-pAb) and 40 μM tetradecapeptide. Internal Ang peptide standards (New England Peptide, Gardner, MA or Anaspec, San Jose, CA, USA) were added, the mixture was diluted with 90% acetonitrile containing 0.3% TFA and spotted onto an MTP 384 ground steel target plate (Bruker Daltonics, Billerica, MA, USA). Mass spectra were obtained using an Autoflex III smartbeam MALDI TOF/TOF instrument (Bruker Daltonics, Billerica, MA, USA). The spectral analysis was performed with proprietary Bruker Flex Analysis and Imaging software.

### Plasma collection

Mice were sacrificed by decapitation using a rodent guillotine. Trunk blood was collected in ice-chilled heparinized tubes. Blood was centrifuged at 10,000 x g for 10 minutes at 4°C. Plasma was separated, aliquoted, and stored at -80°C.

### Urine collection and analysis of creatinine and albumin

Mice were placed individually in metabolic cages for 24h-urine collection with free access to food and water. Protease inhibitors (Roche Diagnostics, Indianapolis, IN, USA) were added to prevent protein degradation. Twenty four-hour urine samples were centrifuged at 3,000 x g for 5 min at 4°C to remove debris. Urinary creatinine was measured using a kit purchased from Quidel (San Diego, CA, USA). Urinary albumin excretion was determined using a Mouse Albumin ELISA Quantitation Set (Bethyl Laboratories, Montgomery, TX, USA). The optical density was measured at a wavelength of 450 nm using a Fusion Packard plate reader (Packard BioScience, Meriden, CT, USA).

### Plasma and urinary PRCP activity

Plasma and urinary PRCP activity were measured using the fluorogenic substrate, 7-Mca-APK(Dnp) (Biomol International, Farmingdale NY, USA) in presence of the ACE inhibitor lisinopril. Plasma (40–100 μg protein) or urine samples (4 μl, 1.3±0.1 μg creatinine) were incubated with 100 μl of the reaction buffer containing 50 mM sodium citrate buffer pH 5, 5 mM ZnCl_2_, 150 mM NaCl, 10 μM lisinopril and 50 μM Mca-APK(Dnp). After incubation at 37°C for 1 hour, fluorescence was measured at 328 nm excitation and 393 nm emission wavelengths using a Fusion Packard plate reader (Packard BioScience, Meriden, CT, USA).

### Data Analysis

The relative mesangial matrix area (RMMA), relative glomerular fibrosis area (RGFA) and tubular or glomerular PRCP staining were quantified using MetaMorph image analysis software (Molecular Devices, Inc., Sunnyvale, CA, USA). All data were expressed as mean ± SEM. Unpaired student’s t-test was used to evaluate the differences between two groups. One-way ANOVA and post hoc Bonferroni’s Multiple Comparison Test compared the difference between more than two groups. A value of p<0.05 was considered statistically significant.

## Results

### Metabolic and cardiovascular parameters

**Fig 1:**
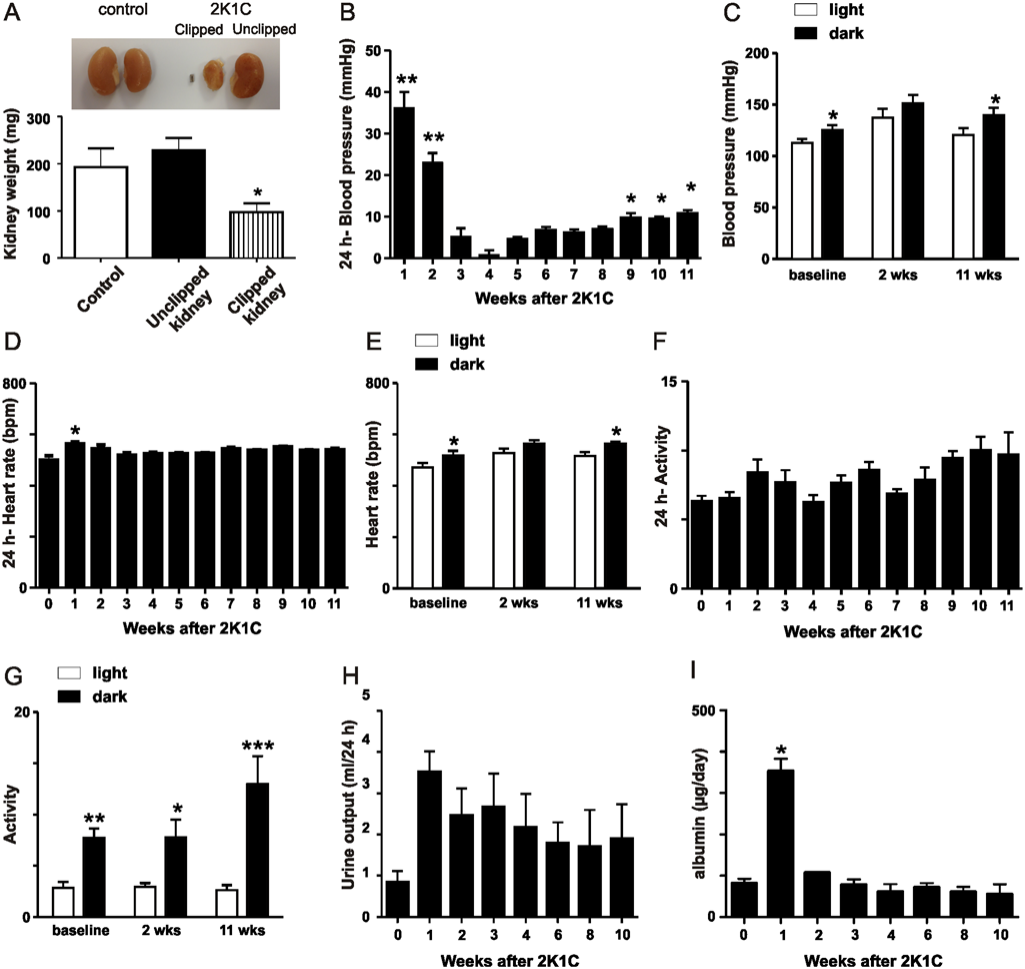
Assessment of kidney weight, blood pressure, heart rate, locomotor activity, urinary output and urinary albumin excretion. A) Kidney weights after 11 weeks of 2K1C. *p < 0.05. B) Weekly blood pressure measurements for 11 weeks after insertion of the renal artery clip. The difference in blood pressure between baseline and after clip placement is shown. *p < 0.001 vs. Control, **p < 0.0001 vs. Control. C) Circadian changes of blood pressure at baseline, 2 weeks and 11 weeks after clip placement. *p < 0.05 vs. light. D) Weekly heart rate measurements for 11 weeks of 2K1C. *p < 0.001 vs. baseline (0 weeks). E) Circadian changes of heart rate at baseline, 2 weeks and 11 weeks after clip placement. *p < 0.05 vs. light. F) Locomotor activity in mice over 11 weeks of 2K1C. G) Circadian changes of activity at baseline, 2 weeks and 11 weeks after clip placement. *p < 0.05, **p < 0.01, ***p < 0.001 vs. light. H) Urinary output at baseline and 1, 2, 3, 4, 6, 8, and 10 weeks after clip placement. I) Daily urinary albumin excretion at baseline and 1, 2, 3, 4, 6, 8, and 10 weeks after insertion of the renal artery clip *p < 0.0001 vs. Control.

After eleven weeks of 2K1C, the kidneys were removed and weighed. Clipped kidneys were significantly smaller and atrophic compared to the contralateral unclipped kidneys and kidneys from control mice (Fig. 1A). As shown in Fig. 1B, a significant rise in blood pressure was observed at one and two weeks after clip placement compared with baseline. The blood pressure returned to baseline levels from weeks three through eight after clip placement, which was followed by a significant increase in blood pressure from baseline at weeks nine, ten, and eleven. There was a significant circadian change of blood pressure at baseline and 11 weeks after 2K1C while at 2 weeks of 2K1C the blood pressure in the 12 h light period was not different from the 12 h dark period (Fig. 1C). A significant increase in heart rate was detected after one week of clip placement compared to baseline (Fig. 1D). Similar to the circadian changes of blood pressure, there was a significant difference of heart rate in the 12 h light period compared to the 12 h dark period at baseline and 11 weeks after 2K1C (Fig. 1E). However, at 2 weeks after 2K1C, the heart rate in the 12 h light period was not different from the 12 h dark period (Fig. 1E). Locomotor activity was not affected by clip placement (Fig. 1F). Circadian changes of activity indicate a significant higher locomotor activity in the 12 h dark period compared to the 12 h light period (Fig. 1G). There was a trend of increased urinary output after clip placement (Fig. 1H) and daily urinary albumin excretion was significantly increased one week after clip placement and returned back to baseline levels during the course of the study (Fig. 1I). The 2-week and 11-week time points were selected for further studies.

### Renal Morphology

**Fig 2:**
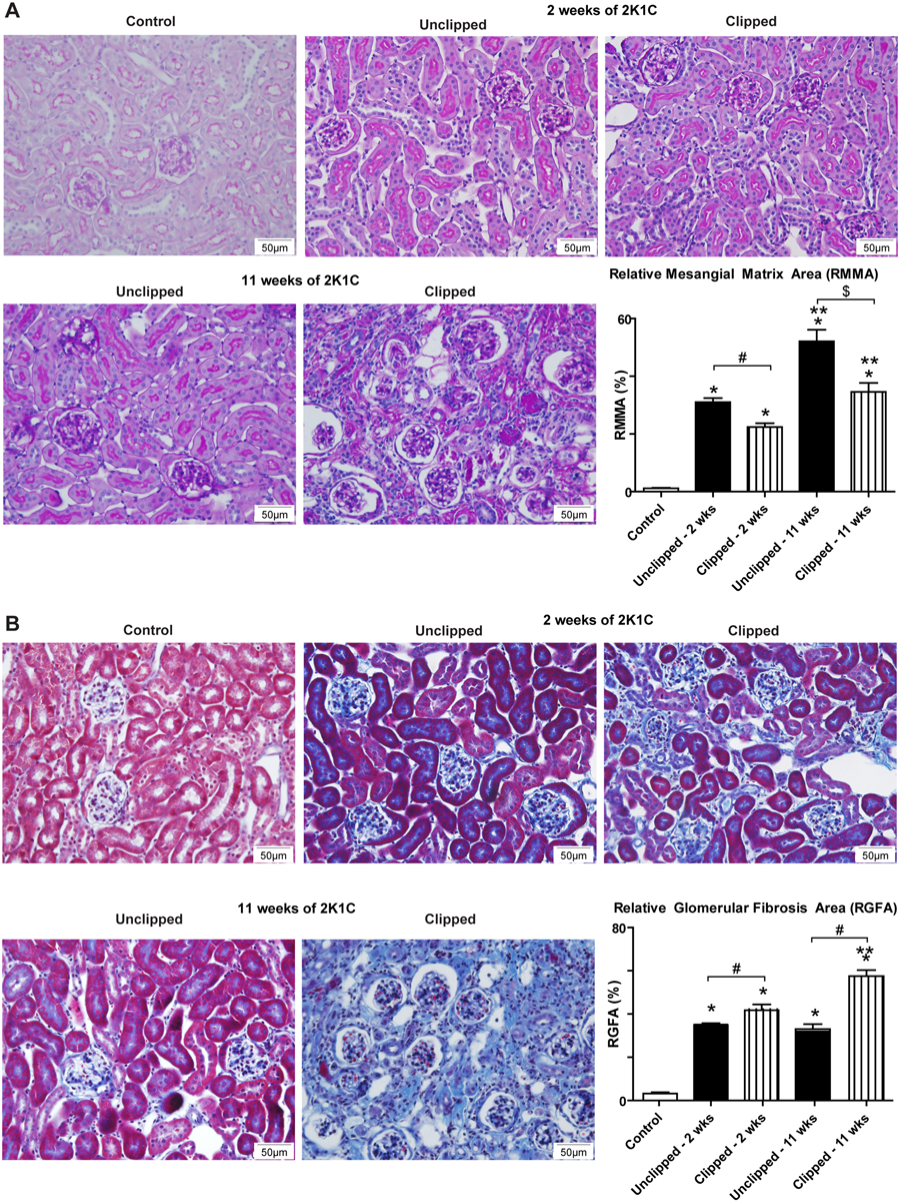
Histological analysis of kidney sections 2 and 11 weeks after renal artery clip placement. A) PAS staining of control, 2K1C unclipped and 2K1C clipped kidneys 2 and 11 weeks after clip placement. B) Masson’s Trichrome staining of control, 2K1C unclipped, and 2K1C clipped kidneys 2 and 11 weeks after clip placement. *p < 0.001 vs. Control, p** < 0.001 vs. 2 wks, ^#^p < 0.001, ^$^p < 0.01.

Mesangial expansion was significantly increased in both 2K1C kidneys at 2 weeks and 11 weeks of 2K1C compared to the control kidneys; however, the unclipped kidneys had a significantly greater mesangial expansion compared to the clipped kidneys at both 2 and 11 weeks post-clipping (Fig. 2A). The clipped and contralateral unclipped 2K1C kidneys also exhibited significantly greater glomerular fibrosis at 2 weeks and 11 weeks of 2K1C compared to the control kidneys. The clipped kidneys showed greater glomerular fibrosis than the unclipped kidneys (Fig. 2B).

### Protein expression of renal PRCP, PREP, and ACE

**Fig 3:**
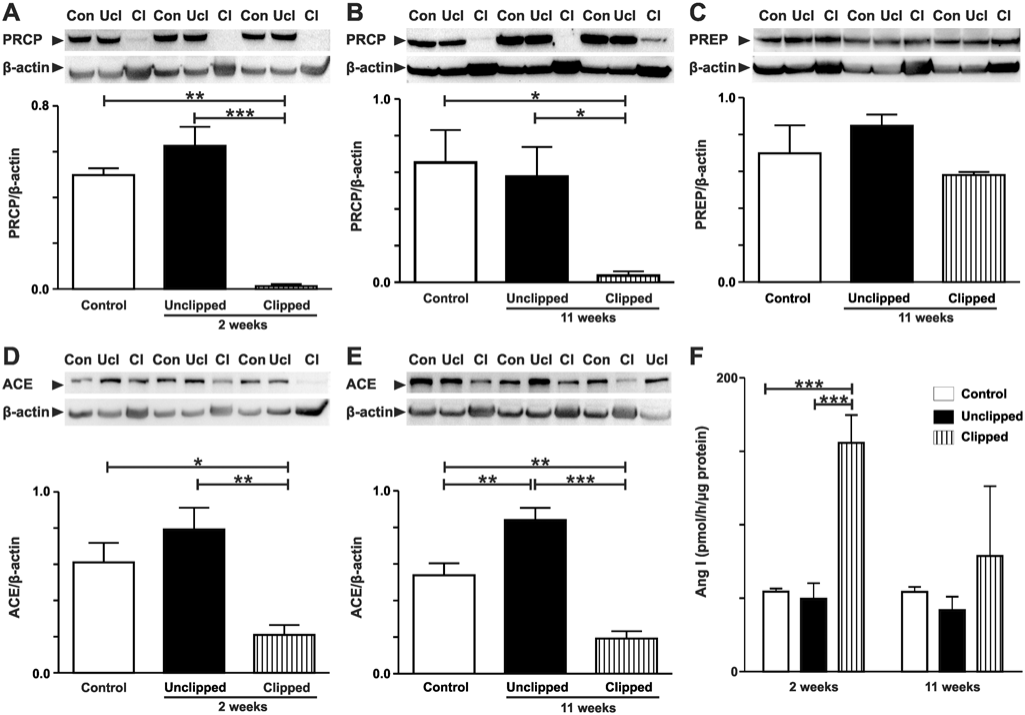
Expression of renal PRCP, PREP, ACE and renin activity 2 and 11 weeks after renal artery clip placement. A) Western Blot of renal PRCP in control, 2K1C unclipped and 2K1C clipped groups after 2 weeks of 2K1C. B) Western Blot of renal PRCP in control, 2K1C unclipped and 2K1C clipped groups after 11 weeks of 2K1C. C) Western Blot of renal PREP in control, 2K1C unclipped, and 2K1C clipped groups after 11 weeks of 2K1C. D) Western Blot of renal ACE in control, 2K1C unclipped, and 2K1C clipped groups after 2 weeks of 2K1C. E) Western Blot of renal ACE in control, 2K1C unclipped, and 2K1C clipped groups after 11 weeks of 2K1C. F) Mass spectrometric analysis of renin activity in control, 2K1C unclipped, and 2K1C clipped groups. For Western blots, protein levels were normalized to β-actin. Values are mean ± SEM. *p < 0.05, **p < 0.01, ***p < 0.001.

Renal PRCP protein expression was significantly reduced in the clipped kidneys but not in the contralateral unclipped kidneys compared to the control kidneys at 2 and 11 weeks after 2K1C (Fig. 3A,B). Expression of renal PREP, an alternative peptidase capable of producing Ang-(1–7), was not changed in both clipped and contralateral unclipped 2K1C kidneys compared to the control (Fig. 3C). Renal ACE expression was significantly increased in unclipped 2K1C kidneys and decreased in clipped 2K1C kidneys compared to controls (Fig. 3D,E).

### Renal renin activity

Renin activity was determined using a MALDI-TOF-based enzyme assays in which kidney homogenates were incubated with the renin substrate tetradecapeptide. Formed Ang I (*m/z* 1296) was absolutely quantified using a stable-isotope labeled peptide standard. Fig. 3F shows that renin activity was significantly increased in clipped 2K1C kidneys compared to control and unclipped kidneys.

### Renal, urinary and plasma PRCP activity

**Fig 4:**
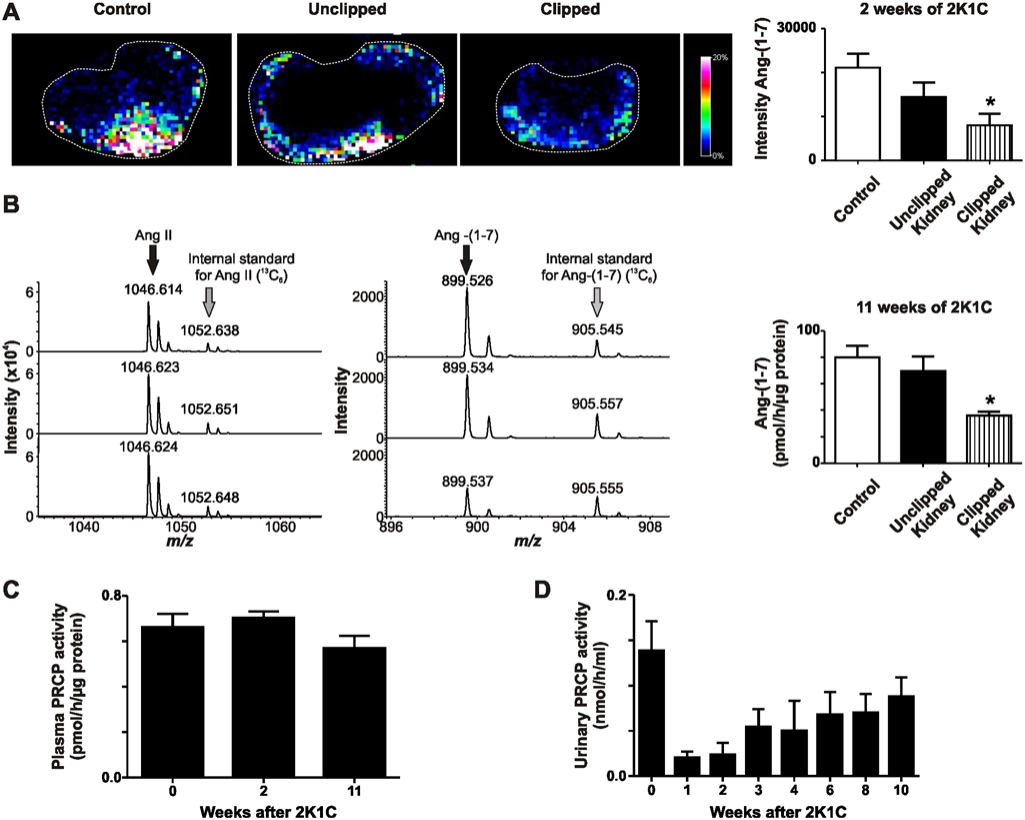
PRCP enzyme activity in control, 2K1C unclipped and 2K1C clipped groups 2 and 11 weeks after clip placement. A) MALDI imaging of Ang-(1–7) formation from Ang II in control kidneys or unclipped and clipped kidney sections obtained after 2 weeks of clip placement. *p < 0.01 vs. Control. B) *In vitro* enzyme activity of Ang-(1–7) formation using homogenates obtained from control kidneys or unclipped and clipped kidneys 11 weeks after renal artery clip placement. *p < 0.01 vs. Control. C) Plasma PRCP activity at baseline and 2 or 11 weeks after clip placement. D) PRCP activity in 24-hour urine samples at baseline and 1, 2, 3, 4, 6, 8, and 10 weeks after insertion of the renal artery clip.

Renal enzymatic activity of PRCP was determined using MALDI-TOF-based enzyme assays that allow for the incubation of kidney sections or homogenates with the natural substrate Ang II. PRCP enzymatic activity in tissue sections was determined *in situ* by detecting the formed product Ang-(1–7) (Fig. 4A). Renal PRCP activity was detected predominantly in the cortex of the unclipped, clipped, and control kidneys and was significantly reduced in the clipped kidneys compared to the control and unclipped kidneys at 2 weeks of 2K1C (Fig. 4A). The *in vitro* enzyme assay confirmed the results obtained with the *in situ* enzyme assay showing a significantly reduced PRCP activity in the clipped kidneys compared to contralateral unclipped and control kidneys after 11 weeks of 2K1C (Fig. 4B). There was no difference in PRCP activity between the contralateral unclipped and control groups (Fig. 4A,B). Plasma and urinary PRCP activities after clip placement were not different from baseline levels (Fig. 4C,D) although there was a trend toward reduced urinary PRCP levels one week after clip placement and a sustained recovery of PRCP levels during the following ten weeks.

### Immunolocalization of renal PRCP

**Fig 5:**
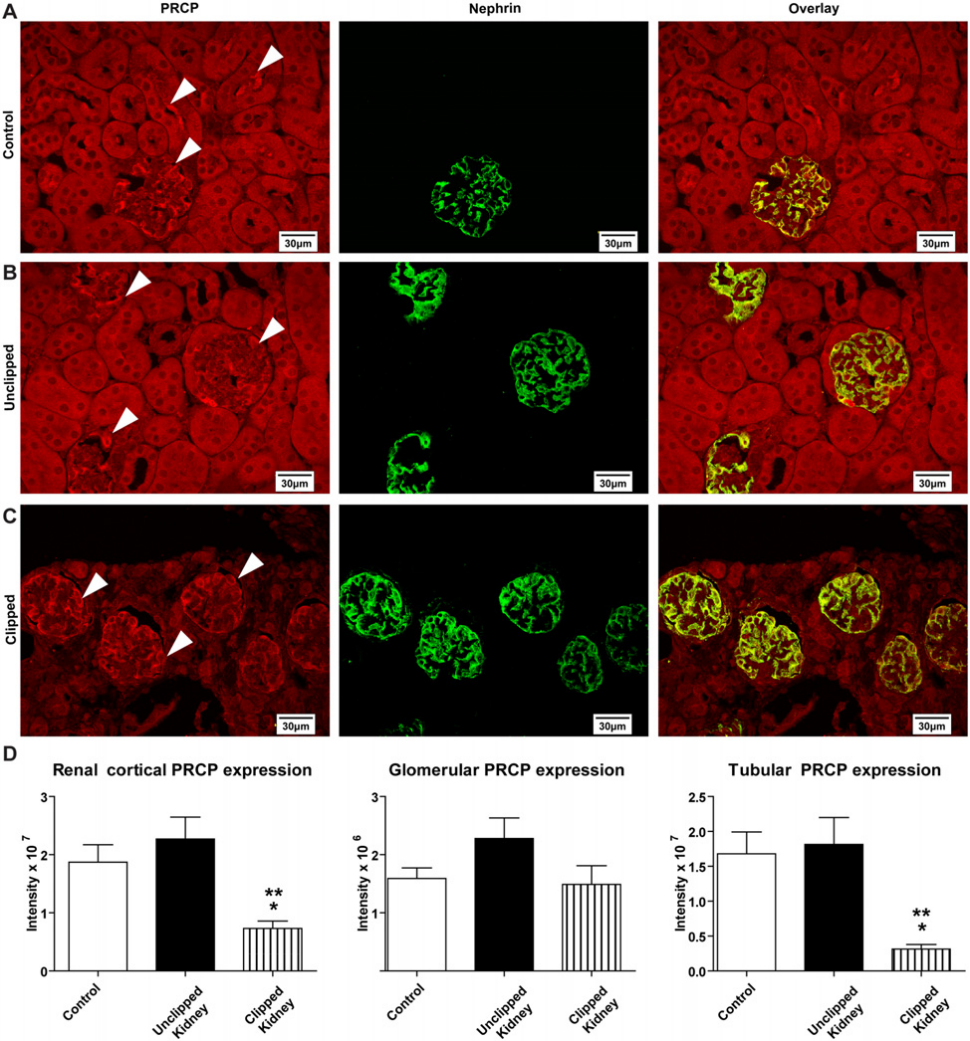
Immunofluorescence analysis of PRCP and nephrin at 60x magnification eleven weeks after 2K1C. White arrows indicate PRCP staining. A) Localization of PRCP and nephrin with overlay in the control kidney. B) Localization of PRCP and nephrin with overlay in the unclipped kidney. C) Localization of PRCP and nephrin with overlay in the clipped kidney. D) Quantitative analysis of cortical, glomerular and tubular PRCP expression in control, unclipped and clipped kidneys. *p < 0.05 vs. Control. **p < 0.01 vs. Unclipped.

Immunofluorescence of PRCP revealed expression of the enzyme in the apical membrane of the renal proximal tubules (Fig. 5A). Furthermore, PRCP co-localized with nephrin in the glomeruli of all groups (Fig. 5A-C). Tubular PRCP expression was reduced in clipped kidneys while glomerular PRCP and nephrin expression were not affected by placement of the renal artery clip as compared with the control (Fig. 5A-C). Overall, glomerular expression of PRCP in the clipped kidneys was unchanged compared to control kidneys and unclipped kidneys while total cortical expression and tubule staining for PRCP was decreased (Fig. 5D).

## Discussion

In this study, the induction of renovascular hypertension caused a significant decrease in clipped kidney weight and an immediate hypertensive response at week one after 2K1C. After one week of clip placement, 24 h blood pressure gradually returned to baseline, showed normal levels at week four post-clipping and increased again toward the end of the study at week 11 after 2K1C. The 24 h heart rate also increased one week after 2K1C and returned to baseline levels during the following ten weeks of 2K1C. Circadian changes were observed at baseline and at 11 weeks after 2K1C. However, at two weeks after 2K1C the 12 h light period did not significantly dip compared with the 12 h dark period although the animals maintained circadian locomotor activity. This finding suggests that, in the early phase, renovascular hypertension disrupts the circadian blood pressure and heart rate rhythm causing a non-dipping effect of the light period.

Previous studies described a gradual increase and full development of hypertension in mice over two weeks and sustained hypertension until week four post-clipping [60–63]. Gradual and sustained hypertension was also observed in rats after clip placement [64,65]. The present study is one of the few studies that monitored the progression of renal artery constriction in mice for longer than four weeks. Consistent with our results, two other studies in mice describe that blood pressure peaked at one week after 2K1C and tended to decline in the following four weeks after 2K1C surgery [66,67] suggesting that progressive stenosis, severe atrophy and complete renal ischemia cause resolution of the hypertension. Similarly, one study in rats demonstrated an immediate increase of blood pressure in the first week of 2K1C that was followed by a decline over the following 4 weeks although levels were still significantly different from baseline at the end of the study [68]. The discrepancies between these studies could be explained by different degrees of renal blood flow restriction, use of other methods to measure blood pressure, the experimental approach, use of different clips and sizes, or the genetic background of the mice.

The 2K1C treatment caused renal injury in both the clipped and the contralateral unclipped kidneys. This was seen with the increased urinary albumin excretion after clip placement although albuminuria normalized within one week of 2K1C following the same trend as observed for blood pressure and heart rate measurements. Indeed, recent studies have shown that many patients with renal impairment are normoalbuminuric, therefore urging a search for underlying pathologies and new markers of CKD [69–72]. The initial increase of blood pressure and urinary albumin excretion in our model may be directly dependent on hyperreninemia and elevated Ang II production especially in the early phase when renin secretion is activated [73] while in the chronic phase, after nine weeks of clip placement, other factors might be responsible for causing the hypertension.

Renal injury was also demonstrated by mesangial expansion and glomerular fibrosis in both kidneys of the clipped animals; however mesangial expansion was exacerbated in the contralateral unclipped group. These renal pathologies are similar to those found in humans with renal artery stenosis where the stenotic kidney showed ischemic changes while the contralateral kidney showed signs of glomerulosclerosis [58]. A separate clinical study involving patients with atherosclerotic renal artery stenosis also demonstrated advanced glomerulosclerosis and similar renal morphology in the affected kidneys [74].

The progression of renovascular hypertension was associated with changes of renal PRCP expression and activity as analyzed using Western blot and mass spectrometry. Renal PRCP expression levels were significantly lower in the clipped kidney compared to both the unclipped and control kidneys. Similar to PRCP, renal ACE2 expression and Ang-(1–7) levels were decreased in 2K1C rats while Ang II was increased suggesting that a differential regulation of the RAS may contribute to hypertension in this model [64]. In addition, ACE was increased in the unclipped kidney while renin was elevated in the clipped kidney confirming previous findings of the RAS in animals with renovascular hypertension [75]. However, protein expression of renal PREP, an alternative Ang-(1–7) forming peptidase, was not different between all groups. These findings suggest that ACE, renin, PRCP and ACE2 participate in renal changes in the 2K1C model and indicate that PREP protein expression is not affected by renal pathologies induced in this model. Indeed, a previous study conducted in our laboratory using genetic deletion mouse models confirmed that the contribution of PREP to renal Ang II processing is negligible compared to that of PRCP and ACE2 [37].

Enzymatic activity studies using established MALDI-TOF mass spectrometric methods [37,38] support the Western blot data. PRCP enzymatic activity was significantly decreased in the clipped kidney compared to the control and unclipped kidney. Although a trend for altered PRCP levels in whole urine was observed, the changes were not significant most likely due to a predominant localization of PRCP in urinary exosomes [76]. Plasma PRCP activity was not changed. Accumulation of Ang II in the 2K1C model could be responsible for decreased renal PRCP activity as Ang II has been shown to inhibit PRCP activity at higher concentrations [77].

To understand and characterize the role of PRCP in the renal cortex during injurious conditions protein expression patterns in the kidney were analyzed using immunofluorescence. PRCP was localized in the renal cortex in the apical membrane of tubules and also within the glomerulus. PRCP expression was significantly reduced in the tubules in the clipped group, perhaps due to the severe tubulointerstitial atrophy present. Severe tubulointerstitial atrophy was also seen in the nephrectomized, affected kidney in patients with atherosclerotic renal artery stenosis [74]. PRCP co-localized with nephrin in all groups, suggesting its expression within podocytes. Expression of nephrin was not affected by the 2K1C approach.

Localization of PRCP to renal tubules and glomeruli fits well with previous studies identifying the renal cortex as the major region in the kidney that generates Ang-(1–7) from Ang II and expresses the Ang-(1–7) binding site [38,78]. We propose that renal Ang II can be degraded to Ang-(1–7) by PRCP in the renal cortex thereby preventing activation of cell signaling through Ang II type I receptors. Therefore, loss of renoprotective PRCP in renovascular hypertension may contribute to progression of renal injury and exacerbate damage in the affected kidney. As one of the primary functions of PRCP in the vasculature is the activation of prekallikrein leading to activated kallikrein and bradykinin release [50,79,80], loss of PRCP in 2K1C could also cause decreased synthesis and secretion of renal kallikrein and thus diminished kinin release. Indeed, the kallikrein-kinin system is reduced in patients and animals with renovascular hypertension [81–83]. Changes in kallikrein and kinin levels could be an alternative explanation for the observed progression of hypertension and renal damage as kinins exhibit vasodilatory effects and may afford renoprotection through AT2 and B2 receptors. Therefore, PRCP may functionally connect the kallikrein-kinin system with the RAS and the combined differential regulation of both systems could contribute to the pathogenesis of renovascular hypertension.
